# Predictors for quality of life in older adults: network analysis on cognitive and neuropsychiatric symptoms

**DOI:** 10.1186/s12877-023-04462-4

**Published:** 2023-12-13

**Authors:** Chaoqun He, Xiangyi Kong, Jinhui Li, Xingyi Wang, Xinqiao Chen, Yuanyi Wang, Qing Zhao, Qian Tao

**Affiliations:** 1 Department of Public Health and Preventive Medicine, School of Medicine, Jinan University, Guangzhou, 510632 China; 2 Division of Medical Psychology and Behaviour Science, School of Medicine, Jinan University, Guangzhou, 510632 China; 3 China–Japan Union Hospital of Jilin University, Jilin University, Changchun, 130031 China; 4 The First Bethune Hospital of Jilin University, Jilin University, Changchun, 130021 China; 5 The First Hospital of Jilin University, Jilin University, Changchun, 130021 China; 6 Neuroscience and Neurorehabilitation Institute, University of Health and Rehabilitation Science, Qingdao, 266071 China

**Keywords:** Network analysis, Mediation, Neuropsychiatric symptoms, Cognition, Attention, Agitation, Quality of life

## Abstract

**Background:**

Quality of life (QoL) of older adults has become a pivotal concern of the public and health system. Previous studies found that both cognitive decline and neuropsychiatric symptoms (NPS) can affect QoL in older adults. However, it remains unclear how these symptoms are related to each other and impact on QoL. Our aim is to investigate the complex network relationship between cognitive and NPS symptoms in older adults, and to further explore their association with QoL.

**Methods:**

A cross-sectional study was conducted in a sample of 389 older individuals with complaints of memory decline. The instruments included the Neuropsychiatric Inventory, the Mini Mental State Examination, and the 36-item Short Form Health Survey. Data was analyzed using network analysis and mediation analysis.

**Results:**

We found that attention and agitation were the variables with the highest centrality in cognitive and NPS symptoms, respectively. In an exploratory mediation analysis, agitation was significantly associated with poor attention (*β* = -0.214, *P* < 0.001) and reduced QoL (*β* = -0.137, *P* = 0.005). The indirect effect of agitation on the QoL through attention was significant (95% confidence interval (CI) [-0.119, -0.035]). Furthermore, attention served as a mediator between agitation and QoL, accounting for 35.09% of the total effect.

**Conclusions:**

By elucidating the NPS-cognition-QoL relationship, the current study provides insights for developing rehabilitation programs among older adults to ensure their QoL.

## Introduction

Rapid population ageing is a global trend, with the number of people aged 60 years and older will be increased to 1.4 billion by 2030 and 2.1 billion by 2050 [1]. Driven by recent declines in fertility/mortality and increases in life expectancy, China is now home to the largest population of older people in the world [2]. The ageing fact raises significant concerns about the well-being of older adults bringing about various challenges involving socio-economic, medical security, and family relationships [1]. Quality of life (QoL) has been recognized by the World Health Organization (WHO) as a key construct to be measured in the older adult population [3], that is an important indicator for successful or poor ageing process [4]. A recent study proposed that the focus of research needs to shift from the treatment of chronic diseases to QoL improvement of the elderly [5]. The QoL could be preserved in older population, provided they remain independence and active, and fulfill social roles [4]. How to ensure QoL has become a pivotal concern of the public and health system. Accumulating evidence of research has identified a variety of predictors for QoL in the ageing group, such as age [6], gender [7], chronic health conditions [8, 9], depression [10, 11], anxiety [12], and functional status [8, 13].

The existing literature suggested that QoL may be used as an outcome measure in aging research across multiple domains, including cognitive disorders, neuropsychiatric conditions, chronic illnesses, physical disabilities, and geriatric interventions [14, 15]. For example, previous studies have used QoL to evaluate the impacts of Alzheimer’s disease on daily function and well-being [16, 17]. In neuropsychiatric research, QoL serves to evaluate symptom severity and treatment effects [18]. Beyond mental health, QoL has also been employed as an indicator of functional status in older adults with chronic diseases [19]. Assessing QoL provides a comprehensive evaluation of how various age-related conditions affect overall well-being and satisfaction with life [20]. The multidimensional nature of QoL makes it a relevant and informative outcome measure in gerontological research.

As people grow older, their cognitive functions gradually decline [21]. The prevalence of cognitive impairment among older adults was substantial. In China, the prevalence of mild cognitive impairment (MCI) and dementia in adults aged 60 years or older was estimated to be 15.5% and 6%, respectively [22]. In the United States, the prevalence of MCI and dementia was 22% and 10%, respectively [23]. Cognitive functions can be divided into several specific domains, including attention, memory, executive function, language, and visuospatial abilities. These different cognitive domains are interrelated and decline with normal ageing [24]. For instance, deficits in attention may impair memory encoding and retrieval processes, limiting an individual’s ability to effectively acquire and recall information [25], while executive function difficulties can affect problem-solving abilities and task switching, making it challenging for individuals to adapt to new situations and effectively handle complex tasks [26]. Meanwhile, cognitive training on one specific cognitive domain showed significant transfer effects on untrained domains [27, 28]. A series of studies showed that cognitive problems can negatively impact on QoL of older people, such as reduced self-care ability in daily life [29], limited social activities and engagement [30], and impaired crucial aspects of daily life [31]. Addressing the cognitive difficulties through appropriate interventions can significantly improve the QoL in older population.

In addition, the older adults is also susceptible to experiencing various neuropsychiatric symptoms (NPS) that can further diminish their QoL [32]. The most common NPS included depression, anxiety, apathy, aggression, and sleep disorder [33–35]. NPS often accompanies cognitive impairment [36]. Indeed, the occurrence of NPS increases in frequency and severity with cognitive decline [37], which is associated with a 3-fold increased risk of dementia and a 2-fold increased risk of MCI [38]. A study has shown that approximately 79.5% of patients with MCI and 91.2% of patients with dementia experience NPS [39]. The NPS not only have a negative impact on QoL of patients, but also impose a great burden on their families and the society [40]. Therefore, effective treatment and management of NPS are also crucial [41].

Easterbrook’s theory proposes that emotional arousal leads to a narrowing of attention and subsequently affects an individual’s perception and experience [42]. According to this theory, when individuals are in an emotionally agitated or highly aroused state, their cognitive resources are restricted and their attention is narrowed. Additionally, previous studies have shown that NPS could have a direct impact on cognitive functions. For example, NPS such as anxiety and depression affect a variety of cognitive functions including working memory [43]. Another study found that hallucinations in MCI individuals were associated with poorer attention, suggesting that NPS may precede cognitive impairment [36]. Other research found that agitation affects the QoL of older people [44]. The existing theory and studies have suggested a path between agitation and attention, which gives us a direction for the exploratory mediation effect. Therefore, an exploratory mediation analysis was performed to test Easterbrook’s theory. We hypothesized that attention mediates the relationship between NPS and QoL.

Traditional analytic approaches rely on total scale scores to describe symptom severity, which may obscure meaningful associations between individual symptoms [45]. In contrast, network analysis has emerged as a novel approach to investigate the interactions among multiple variables and to reveal their complex interrelationships comprehensively [46]. Network model is also useful for understanding the mechanisms of comorbidities [47]. Particularly, network approach has wide applications to understand psychopathology in the field of psychology and psychiatry [48]. In the network, nodes represent symptoms and edges represent correlations between symptoms. The width of the edge indicates the strength of the association between nodes [49]. By applying network analysis, the central symptoms can be identified. The central symptoms have significant clinical value and great practical implication, because the central symptoms are more likely to activate and influence other symptoms, and they are important targets for clinical intervention and treatment [50]. However, network analysis cannot establish causality or directional interconnections among nodes, which can be supplemented by mediation analyses. Mediation analysis can provide an insight into what path of connections among nodes (predictors and mediators) leads to the outcome variables. By exploring NPS-cognition interaction, we can reveal the specific processes that lead to impact on QoL. In addition, mediation analysis can provide preliminary explanations and speculations about the relationships between variables. It can also provide valuable insights for further research and practice. It is noted that network analysis and mediation analysis have different strengths that can complement each other [51]. The combining approach of network and mediation analysis has been widely used in the literature [52–56]. For instance, network and moderator analysis were used to identify what path determines stigma in mental health professionals, considering personality traits, burnout, and professional variables [54].

To this end, our study aims to investigate the relationship between cognitive symptoms, NPS, and QoL in older people with complaints of memory loss using network analysis and mediation analysis. Specifically, we used network analysis to examine the relationship between cognition and NPS, and to identify the most core symptoms. Next, we used an exploratory mediation analysis to investigate the relationship between these core symptoms and QoL. We hypothesized that cognitive symptoms are closely related to NPS, and the core symptom of cognition mediate the relationship between the core NPS and QoL. By elucidating the NPS-cognition-QoL relationship, we hope to provide insights for developing effective interventions and rehabilitation programs for older adults to ensure their QoL.

## Methods

### Participants

We recruited 389 older participants (220 female, mean age = 73.85 years, SD = 8.43) from nursing homes in Nanchang and the psychiatric department of China-Japan Union Hospital in Jilin, China. They were selected based on the following criteria. The inclusion criteria were as follows: (1) aged 60 years and above; (2) self-reported memory decline; (3) native Chinese speaker. The exclusion criteria were as follows: (1) severe visual, auditory, or language impairment that prevented adequate response to assessment questions; (2) severe brain injury or mental illness, such as head injury, tumor, or schizophrenia; (3) history of alcohol or drug dependence or abuse; (4) history of vascular or traumatic brain injury or event that may affect cognitive function; (5) unable to cooperate with the investigation. Sociodemographic indicators, such as age, gender and level of education of the participants, were obtained through self-report. The current study was reviewed and approved by the Ethics Committee of China-Japan Union Hospital (Ethics number: 20,221,020,028), and all participants were asked to provide written consent before initiating the survey. During the data collection process, approximately 20% of the older adults contacted indicated that they did not want to participate in the questionnaire assessment. They were therefore not included in this study.

### Measurements

The Neuropsychiatric Inventory (NPI) [57] was used to assess an individual’s NPS. Previous literature has extensively utilized the NPI scale for evaluating NPS in the general older population [36, 58]. It is a 12-item questionnaire that evaluates both the frequency and severity of the patient’s symptoms and the caregiver’s stress. The 12 items included delusions, hallucinations, agitation, depression/dysphoria, anxiety, apathy, irritability, euphoria, disinhibition, aberrant motor behavior, sleep and nighttime behavior disorders, appetite and eating disorders. The total NPI score is the sum of intensity scores for all 12 items (0 to 144), with higher scores indicating more NPS in patients and greater stress in carers. The Chinese version of the NPI demonstrated good reliability in assessing NPS in dementia patients with Cronbach alpha being 0.84 [59]. The Mini Mental State Examination (MMSE) [60] was used to evaluate an individual’s cognitive functions, including orientation (10 points), memory (6 points), attention (5 points), and language (9 points). Its score ranges from 0 to 30, with lower scores indicating worse cognitive functions. The Chinese version of MMSE has been extensively validated and applied in clinical assessment [61]. We used the 36-item Short-Form Health Survey (SF-36) [62] to assess QoL. The scale has been widely used and is a valid tool for evaluating the health status and QoL of the older adults [63]. The scale consists of 36 items reflecting changes in the patient’s health status over the past year, which included eight dimensions: physical functioning, role physical, bodily pain, general health perception, vitality, social functioning, role emotional, and mental health. Each dimension has a score ranging from 0 to 100, with higher scores indicating better QoL. The Chinese version of the SF-36 scale demonstrates good reliability among older population, making it a suitable tool for assessing their QoL [64, 65].

### Statistical analysis

SPSS 23.0 software was used to conduct descriptive statistical analyses, with means and standard deviations used for continuous variables and frequencies and percentages used for categorical variables. Use Pearson’s correlation for continuous, normally distributed variables, and Spearman’s correlation for ordinal or non-normally distributed data. The prevalence plot of NPS was generated by the R software.

### Network analysis

We conducted a network analysis to investigate the relationship between NPS and cognitive functions using the qgraph, mgm, bootnet, and huge packages in R [66]. To improve the interpretability of the results, we regularized the network using the Least Absolute Shrinkage and Selection Operator method [67]. This method allows the coefficients of non-significant variables to be shrunk to zero, thus achieving the purpose of variable selection. The centrality indices of node strength, closeness, betweenness, and expected influence were calculated using the qgraph package [68]. To determine whether highly central nodes were significantly different from other nodes, we performed a test of differences in node centrality using the bootnet package [66]. Comparison with other nodes allowed us to identify critical symptoms in the network [49]. The mgm package [69] was used to calculate the predictability of each node, which allowed us to assess the extent to which each node could be predicted by its neighboring nodes. In addition, we calculated correlation coefficients between all items in network analysis and QoL.

To ensure the accuracy and replicability of our results, we conducted stability and accuracy tests using the R package bootnet. We assessed the accuracy of network connections and tested whether the centrality estimates differed across variables. We also tested the stability of edge weights and the order of nodes in terms of centrality. To evaluate the stability of centrality indices, we performed bootstrapping test [66] and calculated the correlation stability coefficient. If the stability coefficient exceeded 0.5, centrality was indicated as stable [66].

### Exploratory mediation analysis

We conducted an exploratory mediation analysis [70] to examine the path relationships between variables. It was hypothesized that the core cognitive function mediated the relationship between the core NPS and QoL. A bootstrap mediation analysis was conducted using the PROCESS procedure to test this hypothesis. The data were standardized prior to the mediation analysis. Furthermore, the participants’ gender, age and level of education were included in the analysis as control variables.

## Results

### Descriptive result

**Fig. 1:**
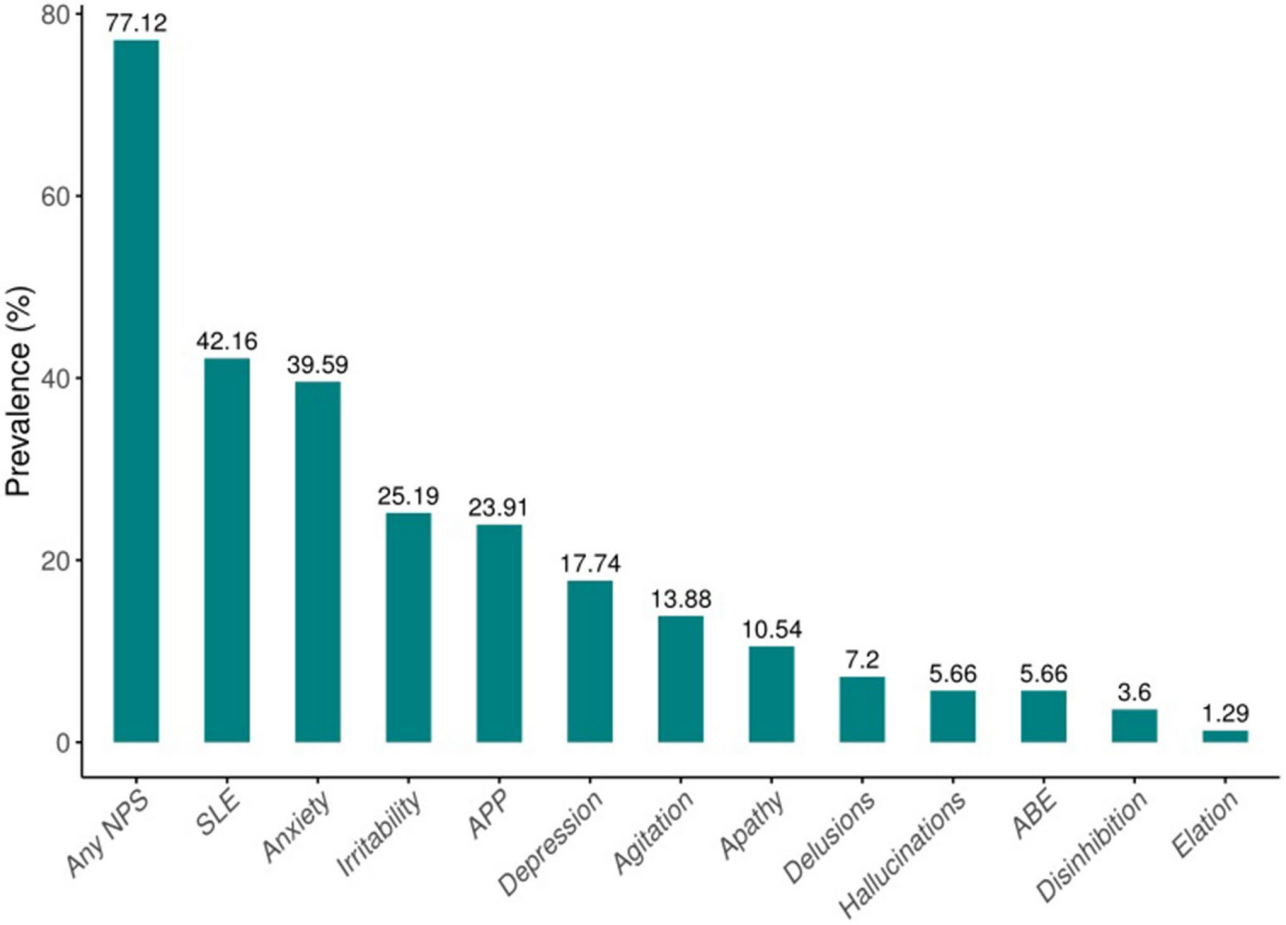
The prevalence of different NPS scores ≥ 1 (n = 389). Note: NPS = Neuropsychiatric symptoms; SLE = Sleep and Nighttime Behavior Disorders; APP = Appetite and Eating Disorders; ABE = Aberrant Motor Behavior

Among the participants, 151 (38.80%) had subjective cognitive decline (SCD), 75 (19.20%) had MCI, and 163 (41.9%) had dementia. The average age of the participants was 73.85 (SD = 8.43), with 56.56% were female. In this study, 77.12% of the participants experienced at least one NPS. The NPS symptoms with a prevalence exceeding 10% were selected for network analysis, which included sleep and nighttime behavior disorders (42.16%), anxiety (39.59%), irritability (25.19%), appetite and eating disorders (23.91%), depression (17.74%), agitation (13.88%), and apathy (10.54%) (Fig. 1). These symptoms were selected based on previous research and clinical significance [71–75]. They are the most prevalent and clinically worrisome neuropsychiatric symptoms among older adults and have received the attention of numerous researchers [35, 76]. The four cognitive domains, including orientation, memory, attention, and language, were selected for network analysis. Demographic information about the participants was listed in Table 1.

**Table 1:** Clinical and demographics data (n = 389)

Variable	Mean (SD) or n (%)
Age (year)	73.85 (8.43)
Gender (women)	220 (56.56)
Education	
Illiteracy	26 (6.68)
Primary school	66 (16.97)
Junior high school	117 (30.08)
High school	80 (20.57)
University or above	100 (25.71)
MMSE total scores	23.31 (5.14)
Cognition domain scores	
Orientation	8.31 (1.92)
Memory	4.07 (1.53)
Attention	3.61 (1.62)
Language	7.31 (1.28)
NPI total scores (mean (P_25_, P_75_))	2.00 (1.00, 8.00)
SF-36 total scores	479.28 (201.02)

Note: Values were expressed as n (%) or mean (SD). The NPI total scores were summarized using the median, along with the 25th and 75th percentiles. Abbreviations: MMSE = Mini-Mental State Examination; NPI = Neuropsychiatric Inventory; SF-36 = 36-item Short-Form Health Survey; SD = Standard Deviation

### Network analysis result

**Fig. 2:**
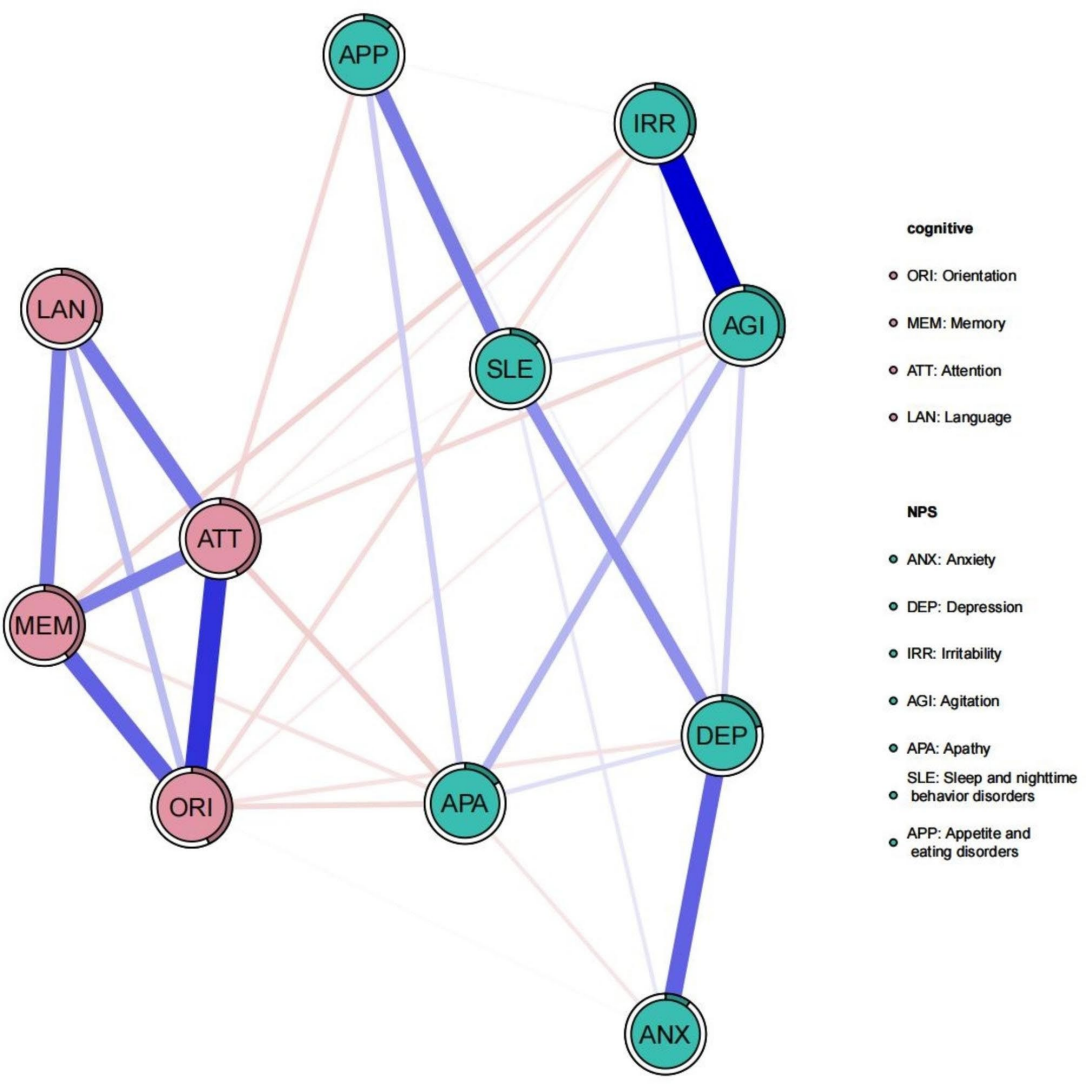
Network of cognition and NPS (n = 389). Note: Nodes represent evaluated variables, and edges represent connections between variables, with the edge width corresponding to the strength of the connections; blue (red) edges represent positive (negative) connections; the circle surrounding each node represents the predictability estimate, which indicates the level to which neighboring nodes can predict a node (similar to R^2^)

**Fig. 3:**
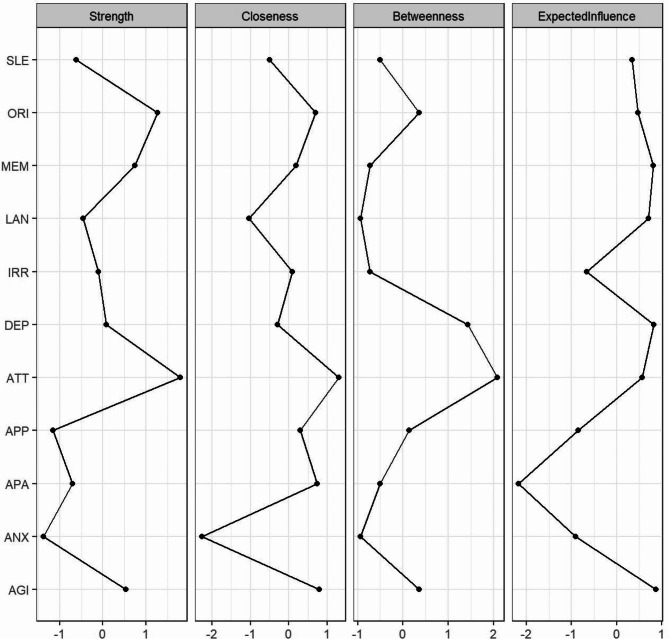
Centrality plot of the cognition and NPS network. Note: The plot is standardized, with higher scores indicating greater centrality. SLE = Sleep and Nighttime Behavior Disorders; ORI = Orientation; MEM = Memory; LAN = Language; IRR = Irritability; DEP = Depression; ATT = Attention; APP = Appetite and Eating Disorders; APA = Apathy; ANX = Anxiety; AGI = Agitation.

As shown in Fig. 2, the relationship between NPS and cognitive function involved several dimensions. The centrality estimates of the network nodes were presented in Fig. 3. “Attention” was the variable with the highest centrality in cognitive functions (strength = 1.79, closeness = 1.30, betweenness = 2.08). In addition, “Attention” demonstrated high predictability (predictability = 0.427), ranking second only to “Orientation” (predictability = 0.429). “Agitation” had the highest centrality and predictability in NPS (strength = 0.54, closeness = 0.79, expected influence = 0.87, predictability = 0.30). Although we did not find connections between the “Language” node and any of the NPS nodes, the “Language” node was strongly associated with the other three cognitive functions (Orientation (*r* = 0.437, *P* < 0.001), Memory (*r* = 0.496, *P* < 0.001), and Attention (*r* = 0.508, *P* < 0.001). The highest correlation between the variables in the network analysis was 0.63 and between the variables in the network analysis and QoL was 0.40 (Table 2). The results of the network analysis revealed good stability with a stability coefficient of 0.67. The stability analysis of the edge weights reliably estimated the strength of the ties within the network. Further, we found that even though up to 50% of the nodes in each network were discarded, their order of centrality remained stable.

**Table 2:** Correlation coefficient of cognitive functions, NPS and QoL

Variables	1	2	3	4	5	6	7	8	9	10	11	12
1. Orientation	1											
2. Memory	0.628^***^	1										
3. Attention	0.590^***^	0.567^***^	1									
4. Language	0.437^***^	0.496^***^	0.508^***^	1								
5. Agitation	-0.256^***^	-0.161^**^	-0.256^***^	-0.126^*^	1							
6. Depression	-0.159^**^	0.005	-0.089	-0.069	0.170^**^	1						
7. Anxiety	-0.092	-0.044	0.006	-0.027	0.019	0.339^***^	1					
8. Apathy	-0.247^***^	-0.243^***^	-0.267^***^	-0.184^***^	0.202^***^	0.105^*^	-0.094	1				
9. Irritability	-0.242^***^	-0.225^***^	-0.218^***^	-0.095	0.504^***^	0.158^**^	0.105^*^	0.143^**^	1			
10. APP	-0.122^*^	-0.067	-0.170^**^	-0.092	0.049	0.149^**^	0.02	0.161^**^	0.128^*^	1		
11. SLE	-0.081	-0.013	-0.119^*^	-0.027	0.128^*^	0.280^***^	0.181^***^	0.049	0.111^*^	0.259^**^	1	
12. QoL	0.396^***^	0.385^***^	0.395^***^	0.403^***^	-0.249^***^	-0.344^***^	-0.347^***^	-0.257^***^	-0.266^***^	-0.211^***^	-0.310^***^	1

Note: ^*^P < 0.05, ^**^P < 0.01, ^***^P < 0.001. APP = Appetite and Eating Disorders; SLE = Sleep and Nighttime Behavior Disorders; QoL = Quality of Life

### Exploratory mediation analysis result

Based on network analysis results, we further examined the relationship between agitation, attention, and QoL. The findings revealed significant correlations among agitation, attention, and QoL. Specifically, agitation showed a significant negative correlation with attention (*r* = -0.256, *P* < 0.001) and QoL (*r* = -0.249, *P* < 0.001). Furthermore, attention exhibited a significant positive correlation with QoL (*r* = 0.340, *P* < 0.001) (Table 2).

Mediation analysis is a statistical method for testing causality [77], but its interpretation of causality is only valid if the direction of influence between variables is clear and all other potentially confounding variables are considered. In the exploratory mediation analysis of the present study, we hypothesized that agitation influences attention rather than attention affects agitation. It is worth noting that the latter hypothesis may also be plausible, and we have therefore taken it into account in interpreting the results of this study (see Discussion).

**Fig. 4:**
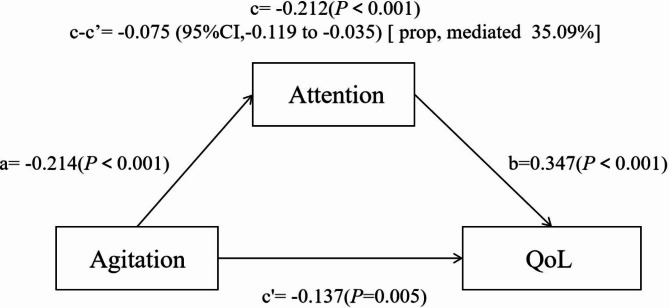
Mediating effect of attention in the relationship between agitation and QoL (n = 389). Note: Quality of life (QoL) was assessed as the dependent variable using the 36-item Short-Form Health Survey (SF-36). The numbers next to the arrows demonstrate the standardized path coefficients. The solid arrows represent the statistically significant paths

The exploratory mediation model (Table 3; Fig. 4) aims to clarify the relationship between agitation as the independent variable and QoL as the dependent variable, by adding a third theoretical variable (attention) as a mediator. The findings showed that attention partially mediated the relationship between the QoL and the agitation. Agitation was a negative predictor of attention (*β* = -0.214, *P* < 0.001), indicating that older people with higher agitation levels were associated with poorer attention. The direct effect of agitation on QoL was negatively significant after controlling for the effect of attention (*β* = -0.137, *P* = 0.005). The indirect effect of agitation on the QoL through attention was significant (95% CI [-0.119, -0.035]). The mediation analysis revealed that attention mediated the relationship between agitation and QoL, accounting for 35.09% of the total effect.

**Table 3:** Exploratory mediation of attention in the relationship between agitation and QoL (n = 389)

Dependent variable	Predictor variable		95% CI	
		*β*	LL	UL
Attention	Agitation	-0.214^***^	-0.309	-0.12
QoL	Attention	0.347^***^	0.248	0.445
QoL	Agitation	-0.137^**^	-0.232	-0.042
QoL	Agitation through attention	-0.075^a^	-0.119	-0.035

Note: ^**^P < 0.01, ^***^P < 0.001. QoL = Quality of Life; CI = Confidence Interval; *β* = Standardization Coefficient; LL = Lower Limit; UL = Upper Limit; ^a^ Overall indirect effect. The analysis was adjusted for gender, age, and education level

## Discussion

To our knowledge, this is the first study to use network analysis and mediation analysis to investigate the relationship among NPS, cognitive function, and QoL in older population. Our findings revealed that NPS had a prevalence of 77.12% among the participants. Furthermore, the occurrence and severity of NPS increased as cognitive decline worsened, while the QoL decreased. These results are in line with previous research findings [78, 79].

The network analysis in this study visualized the complex network of relationships between different NPS and cognition, providing important insights into their interaction. We observed that “Attention” had the highest values in three centrality measures (strength, closeness, and betweenness) among cognitive functions, indicating its crucial role in the network. The importance of attention in cognition has been widely reported in the literature [80, 81]. It reflects an individual’s ability to select, focus, and sustain attention to external stimuli. Whatever its severity, attention disorders have an impact on other cognitive functions (such as memory and learning). Most importantly, it has the potential to perturb execution of daily activities and skills [82].

In NPS, “Agitation” has the highest values in the centrality measures of strength, closeness, and expected influence, indicating that it is the most important in the network. Moreover, there is a strong correlation between agitation and symptoms such as irritability and apathy, which increases its centrality in the network. On the other hand, the higher centrality of agitation aligns with its significance in psychology and neuroscience. For example, studies have shown the widespread presence of agitation and aggressive behavior NPS [83]. Therefore, it is reasonable for the “Agitation” to have higher centrality in the network. Interestingly, the “Language” node was not directly linked to the NPS node, but it exhibited a robust and meaningful relationship with the other three cognitive functions. These results suggested that language function may indirectly affect NPS by influencing other cognitive functions, which provides clues for further exploration of the role of language function in the development of NPS.

The results of the network analysis revealed the importance of variables within the network and provided clues to further understanding of the relationship between cognitive function and NPS. Exploratory mediation analysis revealed the mediating role of attention between agitation and QoL, suggesting that agitation indirectly affects QoL by influencing attention. This finding supports Easterbrook’s theory, which believes that emotional arousal leads to a narrowing of attention and affects an individual’s perceptions and experiences [42]. Agitation may impact various aspects of attention. First, agitation can make it difficult for individuals to concentrate because they become emotionally agitated and their attention is disrupted. Second, it may cause individuals to focus excessively on potential threats or conflicts, leading to a bias towards negative or hostile stimuli while ignoring other important information [84]. Additionally, agitation can interfere with the regulation of attention in social interactions, making it difficult to recognize and understand the emotions and intentions of others [85]. Furthermore, limited attention has a negative impact on individuals’ QoL [86]. These attention difficulties can manifest across various domains of daily life, including work, learning, social interactions, and emotional regulation, consequently reducing overall life quality. In individuals with Attention-Deficit/Hyperactivity Disorder (ADHD) [87, 88], Post-Traumatic Stress Disorder (PTSD) [89], and Autism Spectrum Disorder (ASD) [90, 91], it is commonly observed that attention problems, impulse control difficulties, agitation/aggressive, and anger-related issues coexist, indicating a link between agitation and attention. It is necessary to mention that the mechanisms of influence in these areas are not fully understood from the intervention point of view. Our findings are consistent with previous studies [92], indicating that a significant portion of the shared variance between agitation and cognition can be explained by attention. Therefore, it may be possible to attempt to alleviate the effects of agitation on QoL by intervening on attention. For instance, cognitive training may be used to help older adults learn how to better regulate their attention, focusing on the positive and reducing the concern for negative agitation. Psychological and social support may also be provided to older adults to help them deal effectively with agitation and change the way agitation is perceived. Strengthening social support for older adults and providing more social resources and emotional support can help to distract attention and reduce the impact of agitation on QoL. It is important to emphasize that the results of this study only provide preliminary basis for the development of possible interventions, and the design and implementation of specific interventions need to be considered in conjunction with the results of further studies and practical situations.

It is worth noting that the mediation analysis in this study were conducted based on cross-sectional data, so it is possible that alterations in attention in older adults may also affect agitation. Actually, in another set of exploratory mediation analysis, we found that agitation mediated the relationship between attention and QoL, although this mediating effect was only 8.30%. These findings suggest that inferring causal direction from mediation analysis in cross-sectional studies is difficult. In addition, unmeasured other variables related to attention and QoL may influence the observed mediating effects. Longitudinal studies that measure data at multiple time points are the only way to derive causal associations. However, our findings suggest an important role for attention in the effects of agitation on QoL.

### Limitations and future directions

This study has several limitations. First, the use of a cross-sectional design prevents us from establishing causal relationships. Future studies could be conducted longitudinally to track changes in cognitive function and NPS over time in older adults, which could help us to investigate trends in the dynamics of cognitive decline and its predictive factors at different stages, and reveal causal relationships between variables. Second, the cognitive assessment dimension includes only four measures from the MMSE scale and does not encompass other cognitive dimensions such as executive function and visuospatial abilities. Future research could consider incorporating these cognitive dimensions into analysis as well. Third, we adjusted for a limited set of covariates in the mediation analysis, including gender, age, and education level, while ignoring other potential confounders, such as marital status and living area. These potential confounders were not controlled for due to data unavailability. Future research could collect data on more covariates and perform more comprehensive analyses. Fourth, participants in this study were recruited from a nursing home and an outpatient clinic in two regions, which may not be representative of the older population across different regions of China. Therefore, the generalizability and applicability of our findings need to be further examined in more diverse older populations. Fifth, the network analysis found that there may be various other mediations going through attention which is picked up in the high betweenness score, but exploring the associations among these variables was beyond the scope of our study. Future studies could further explore this interesting issue. The last but not the least, the sample in this study was population specific and future researches could use network analysis to explore the relationship between NPS and cognitive function in groups with normal/SCD, MCI and dementia. These different groups may have different symptom networks related to the severity and subgroups of cognitive impairment.

## Conclusions

In conclusion, a network model of the relationship between NPS and cognitive functions was developed in this study. We identified the crucial roles of agitation and attention in the network, and their impact on QoL. We found that attention mediated the relationship between agitation and QoL, accounting for 35.09% of the total effect. This suggests that attention plays a critical role in the mechanism of how agitation affects QoL. These findings provide valuable insights into understanding the associations among NPS, cognition, and QoL in older adults, with significant clinical implications.

## Data Availability

The data may be available from the corresponding author on reasonable request.

## Abbreviations

QoL: Quality of Life
WHO: World Health Organization
NPS: Neuropsychiatric Symptoms
NPI: Neuropsychiatric Inventory
MMSE: Mini Mental State Examination
MCI: Mild Cognitive Impairment
SCD: Subjective Cognitive Decline
SF-36: 36-item Short-Form Health Survey
SD: Standard Deviation
ADHD: Attention-Deficit/Hyperactivity Disorder
PTSD: Post-Traumatic Stress Disorder
ASD: Autism Spectrum Disorder
LL: Lower Limit
UL: Upper Limit
CI: Confidence Interval
ABE: Aberrant Motor Behavior
SLE: Sleep and Nighttime Behavior Disorders
ORI: Orientation
MEM: Memory
LAN: Language
IRR: Irritability
DEP: Depression
ATT: Attention
APP: Appetite and Eating Disorders
APA: Apathy
ANX: Anxiety
AGI: Agitation
